# UniCAR T-Cell Potency—A Matter of Affinity between Adaptor Molecules and Adaptor CAR T-Cells?

**DOI:** 10.3390/ijms25137242

**Published:** 2024-06-30

**Authors:** Hugo Boutier, Liliana R. Loureiro, Lydia Hoffmann, Claudia Arndt, Tabea Bartsch, Anja Feldmann, Michael P. Bachmann

**Affiliations:** 1 Department of Radioimmunology, Institute of Radiopharmaceutical Cancer Research, Helmholtz-Zentrum Dresden-Rossendorf (HZDR), 01328 Dresden, Germany; h.boutier@hzdr.de (H.B.); l.loureiro@hzdr.de (L.R.L.);; 2 Mildred Scheel Early Career Center, Faculty of Medicine Carl Gustav Carus, TU Dresden, 01307 Dresden, Germany; 3 National Center for Tumor Diseases Dresden (NCT/UCC), German Cancer Research Center (DKFZ), 69120 Heidelberg, Germany; 4 Faculty of Medicine and University Hospital Carl Gustav Carus, Technische Universität Dresden, 01307 Dresden, Germany; 5 German Cancer Consortium (DKTK), Partner Site Dresden, and German Cancer Research Center (DKFZ), 69120 Heidelberg, Germany

**Keywords:** adaptor CAR platform, UniCAR T-cells, E5B9 UniCAR epitope, cancer immunotherapy, Fibroblast Activation Protein (FAP)

## Abstract

Although Chimeric Antigen Receptor (CAR) T-cells have shown high efficacy in hematologic malignancies, they can cause severe to life-threatening side effects. To address these safety concerns, we have developed adaptor CAR platforms, like the UniCAR system. The redirection of UniCAR T-cells to target cells relies on a Target Module (TM), containing the E5B9 epitope and a tumor-specific binding moiety. Appropriate UniCAR-T activation thus involves two interactions: between the TM and the CAR T-cell, and the TM and the target cell. Here, we investigate if and how alterations of the amino acid sequence of the E5B9 UniCAR epitope impact the interaction between TMs and the UniCAR. We identify the new epitope E5B9L, for which the monoclonal antibody 5B9 has the greatest affinity. We then integrate the E5B9L peptide in previously established TMs directed to Fibroblast Activation Protein (FAP) and assess if such changes in the UniCAR epitope of the TMs affect UniCAR T-cell potency. Binding properties of the newly generated anti-FAP-E5B9L TMs to UniCAR and their ability to redirect UniCAR T-cells were compared side-by-side with the ones of anti-FAP-E5B9 TMs. Despite a substantial variation in the affinity of the different TMs to the UniCAR, no significant differences were observed in the cytotoxic and cytokine-release profiles of the redirected T-cells. Overall, our work indicates that increasing affinity of the UniCAR to the TM does not play a crucial role in such adaptor CAR system, as it does not significantly impact the potency of the UniCAR T-cells.

## 1. Introduction

Over the past decades, various strategies have been implemented to reprogram immune cells against tumor cells and more recently against autoreactive immune cells [1,2,3,4,5,6,7,8]. Among them, Chimeric Antigen Receptor (CAR) T-cells have shown promising clinical results in hematologic malignancies, leading to the approval of several CAR T-cell therapies since 2017 [1,2,3,4,5,6,7,8,9,10,11,12,13]. Despite their remarkable efficacy, CAR T-cells can cause severe to life-threatening adverse events including Cytokine Release Syndrome (CRS), neurotoxicity and on-target/off-tumor effects [14,15,16,17,18,19]. In order to address these safety issues, we and others have developed modular CAR platforms [20,21,22,23,24,25,26,27,28,29,30,31,32,33,34,35,36,37,38]. In these adaptor CAR systems, T-cells are engineered to express a universal CAR receptor that does not interact directly with a Tumor-Associated Antigen (TAA) or Tumor-Specific Antigen (TSA). Instead, the genetically modified T-cells are redirected to target cells by bifunctional adaptor molecules.

A promising example of such an adaptor CAR platform is the Universal CAR system UniCAR [25,26], which is based on two distinct components: UniCAR T-cells and adaptor molecules named Target Modules (TMs). The extracellular single-chain Fragment variable (scFvs) binding domain of the UniCAR, which is derived from the anti-La (αLa) monoclonal antibody (mAb) 5B9 [39,40,41], is not directed to a TAA. Instead, the UniCAR recognizes the peptide epitope E5B9, with the amino acid (aa) sequence KPLPEVTDEY [25,26,42], which is part of the primary aa sequence of the human nuclear La/SS-B autoantigen (aa 95-104) [25,42,43,44]. Such a peptide sequence was selected for the UniCAR system as it is cryptic in the native La protein and no immune responses to this epitope have been seen in autoimmune patients [25,45]. We therefore expect it not to be immunogenic if applied to patients with tumors or systemic autoimmune diseases such as SLE or primary Sjögren’s syndrome.

In order to develop a novel TM, the E5B9 peptide epitope has to be fused to an anti-TAA binding moiety. As the activation of the UniCAR T-cells relies on the cross-linkage of the TMs to the target and effector cells, the engineered T-cells are not harmful to healthy tissues in the absence of a TM. In addition, a broad variety of antigens can be targeted using TMs of different specificities without the need to re-engineer the T-cells. Moreover, different structures can be used for TM design, including small-molecules [46], nanobodies [47], scFvs [48] and Ig-related recombinant antibodies [48,49]. By controlling the specificity, half-life, valency and concentration of the UniCAR TMs, UniCAR T-cell potency can be modulated for better safety, controllability and flexibility [20,22,25,26]. 

In recent years, there has been growing interest in the modification of conventional CAR T-cells to improve their efficacy and safety [50,51,52,53,54,55,56,57,58,59,60,61,62,63]. The addition of suicide genes is a promising example of a safety strategy that allows for the selective elimination of engineered T-cells if severe toxicities occur. Mestermann et al. describe a pharmacological regulation of the active CAR T-cells using kinase inhibitors. Abken et al. presented the idea of including additional genes into the CAR construct encoding proinflammatory cytokines to modulate the tumor microenvironment. Furthermore, logical gating is proposed to further improve the safety of CARs [21,26,55]. In addition, CAR genes themselves have been modified. Several parameters, including the affinity of the antigen-binding domain to its target, the distance between the antigen-binding domain and the membrane of the engineered T-cells, the selection of signaling and costimulatory domains, and the modulation of downstream activation signaling, have been shown to have a substantial impact on the therapeutic outcome of CAR T-cells [55,56,57,58,59,60,61,62,63]. In particular, modifying the CAR affinity has a direct impact on the activation and functions of CAR T-cells [58,63]. 

In the context of modular CAR platforms, all the parameters responsible for the potency of such adaptor systems are not fully understood. The redirection of CAR T-cells to target cells depends on two interactions with two different affinities, between (i) the TM and the CAR T-cell or (ii) the TM and the target cell. So far, adaptor molecules have been developed empirically, i.e., discovered and optimized through experimentation rather than designed on the basis of a comprehensive theoretical understanding. In addition, the literature has mainly focused on the affinity of the adaptor molecule to the selected TAA and little is known about the influence of the affinity of the adaptor to the adaptor CAR on the potency of adaptor CAR T-cells. This interaction can simply be tuned by modifying the part of the bridging molecule binding to the CAR without the need to re-engineer the CAR, as recently demonstrated with the split, universal and programmable (SUPRA) CAR system [21]. The same is true for the previously described adaptor CAR platform UniCAR, for which not only proof of functionality and controllability has been shown in a series of preclinical studies [20,22,25,26,46,47,48,49,64] but even in first phase 1 clinical trials [65] (NCT04230265, NCT04633148). 

Here, we investigate whether and how the interaction between UniCAR TMs and UniCAR T-cells influences the functionality and/or potency of the UniCAR T-cells. For this purpose, we screened and analyzed the contribution of each aa of the UniCAR epitope on the affinity of the mAb 5B9 to the E5B9 peptide. After estimating how the aa flanking the E5B9 epitope in the native La protein may affect the interaction between the mAb 5B9 and UniCAR epitope, we identified an aa sequence, named E5B9L, to which the mAb 5B9 had the highest affinity. Previously established UniCAR TMs directed to the Fibroblast Activation Protein (FAP) [48] were then engineered to integrate the modified E5B9L epitope. Finally, we assessed the impact of such a modification on the potency of UniCAR T-cells.

## 2. Results

### 2.1. Binding Studies of the αLa 5B9 mAb to La-Derived Synthetic Peptides

**Figure 1:**
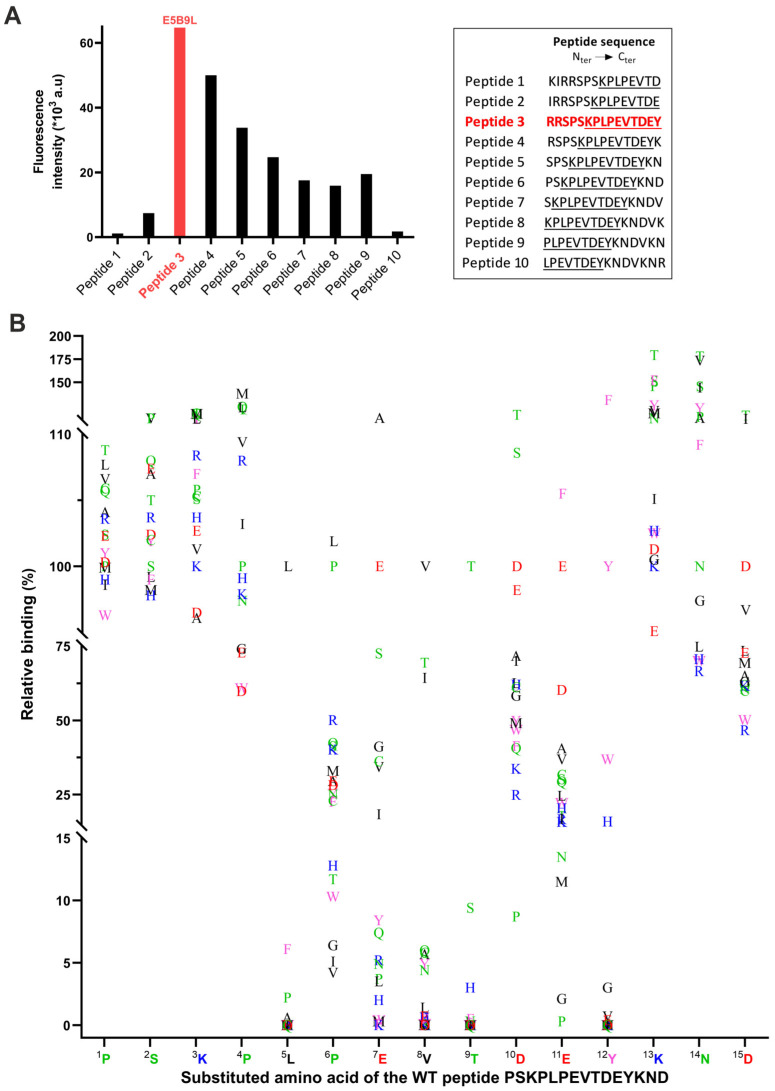
Affinity study of αLa mAb 5B9 to La-derived synthetic peptides. The affinity of αLa mAb 5B9 to La-derived peptides was performed by PEPperMAP^®^ epitope mapping. (**A**) Peptides with a peptide–peptide overlap of 14 aa were generated from the La protein. E5B9L sequence (“Peptide 3”) is indicated in bold and red. The 10 aa from the E5B9 peptide epitope are underlined. (**B**) Each aa of the wild-type peptide PSKPLPEVTDEYKND was replaced by the 20 standard aa, generating 300 peptides. The relative binding in percentage of the αLa mAb 5B9 to each of these peptides compared to the WT peptide is shown as a function of each substituted aa. Amino acids with different properties are color-coded as follows: polar (green), non-polar (black), aromatic (pink), negatively charged (red), and positively charged (blue).

In order to learn whether or not the interaction between the antibody-binding domain of UniCARs and the peptide epitope of the adaptor TM directly influences the effector functions of UniCAR T-cells, we first investigated the binding properties of the αLa mAb 5B9 through epitope mapping studies. We therefore assessed the binding of the αLa mAb 5B9 to 15-aa-long peptides overlapping by 14 aa that were derived from the primary aa sequence of the native La/SS-B protein (Figure 1A). In agreement with our previous epitope mapping results [25], all the overlapping peptides containing the minimal E5B9 epitope sequence KPLPEVTDEY are recognized by the αLa mAb 5B9 (Figure 1A, peptides 3 to 8). Peptide 9 is an exception: it contains the aa PLPEVTDEY and thus lacks the N-terminal lysine residue of the previously estimated minimal E5B9 epitope but is still recognized by the αLa mAb 5B9. This can be taken as a hint that this lysine residue is not essential for the binding of the αLa mAb 5B9. However, the data also show that all peptides (Figure 1A, peptides 3 to 6) containing two or more additional aa upstream of the N-terminus of the minimal epitope have an improved binding, suggesting their potential to enhance the binding capability of the αLa mAb 5B9. The best binding of the αLa mAb 5B9 was observed for the peptide variant RRSPSKPLPEVTDEY (the minimal epitope sequence is underlined) hereafter referred to as E5B9L (Figure 1A, peptide 3). Moreover, there is a dramatic decrease in binding of the αLa mAb 5B9 to peptide 2 (IRRSPSKPLPEVTDE) lacking the C-terminal tyrosine residue of the minimal epitope compared to peptide 3 (RRSPSKPLPEVTDEY), underlining the relevance of this single tyrosine residue in this epitope sequence.

In order to learn which of the aa plays a major role in the binding of the αLa mAb 5B9 to the peptide epitope and which ones can be neglected, we studied the binding capability of the αLa mAb 5B9 to peptides obtained by replacing each aa residue of the wild-type peptide PSKPLPEVTDEYKND (minimal epitope underlined) with one of the 20 standard aa. The results of all substitutions are presented in Figure 1B. This study confirms the importance of the tyrosine residue in the binding of the αLa mAb 5B9 to La-derived peptides (Figure 1B, ^12^Y): ^12^Y can only be replaced by another aromatic aa, while all other aa replacements result in an almost complete loss of the binding of the αLa mAb 5B9. Interestingly, the replacement of the ^12^Y residue by phenylalanine even improves the binding of the antibody while tryptophane reduces the binding. Similarly, we observe that the leucine (Figure 1B, ^5^L) and the threonine residues (Figure 1B, ^9^T) exhibit an essential character as their replacement leads to a significant decrease in relative binding. The threonine (Figure 1B, ^9^T) cannot even be replaced by a serine residue. In addition, the replacement of the valine (Figure 1B, ^8^V) was poorly tolerated, highlighting its key role in the binding of the antibody. In contrast, all replacements of aa at positions 1 to 3 (Figure 1B, ^1^P, ^2^S, ^3^K) were tolerated. Also, the replacement of the second proline (Figure 1B, ^4^P) was widely acceptable. As already mentioned for the possible replacement of ^12^Y with phenylalanine, the substitution of some aa could even improve the binding. For example, the substitution of ^13^K and ^14^N with a threonine, respectively, improved the binding of the αLa mAb 5B9 to the epitope (Figure 1B).

### 2.2. Design of αFAP-E5B9L TMs

**Figure 2:**
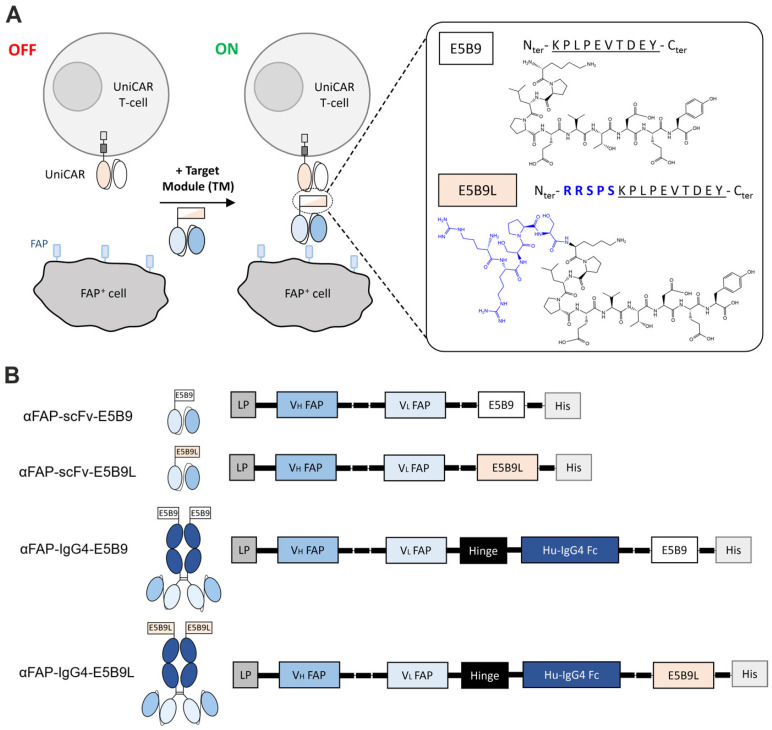
Design of αFAP TMs for redirection of UniCAR T-cells to FAP^+^ target cells. (**A**) Schematic representation of the UniCAR system and αFAP TMs targeting the FAP antigen. UniCAR T-cells are second generation CARs composed of an extracellular scFv targeting the peptide epitope E5B9, along with CD28 transmembrane and intracellular signaling domains. The presence of an adaptor target module (TM), i.e., αFAP TM, containing either E5B9 or E5B9L peptide, is required to redirect UniCAR T-cells to FAP^+^ cells and trigger effector cell functions. (**B**) Different formats of αFAP TMs were generated: scFv TMs and IgG4-based TMs. Both formats contain either the E5B9 peptide epitope or the E5B9L variant. LP: Igκ leader peptide; V_H_: variable domain of the antibody heavy chain; V_L_: variable domain of the antibody light chain; Fc: fragment crystallizing; His: hexa-histidine tag.

According to the results obtained from the binding studies of the αLa mAb 5B9 to La-derived synthetic peptides, we expected that TMs based on either E5B9 or E5B9L should have different binding affinities and would therefore be suitable to learn whether or not higher affinity of the UniCAR to the peptide epitope could influence the functionality of UniCAR T-cell efficiency. Recently, we described the development of scFv- and IgG-based novel TMs for redirection of UniCAR T-cells to FAP, a marker of the tumor microenvironment (TME) [48]. In order to experimentally compare TMs targeting the same TAA with the same FAP-specific antibody-binding domain but having different binding properties to the UniCAR, we developed the respective scFv- and IgG4-based αFAP TMs containing the E5B9L peptide epitope instead of the minimal E5B9 epitope (Figure 2A,B). The substitution of the E5B9 peptide in the previously established monovalent scFv TM αFAP-scFv-E5B9 and bivalent IgG4-based TM αFAP-IgG4-E5B9 [48] by the E5B9L peptide led to the generation of the novel αFAP-scFv-E5B9L and αFAP-IgG4-E5B9L TMs, as schematically summarized in Figure 2B.

### 2.3. Biochemical Characterization and Binding Evaluation of αFAP-E5B9L TMs to UniCAR T-Cells

**Figure 3:**
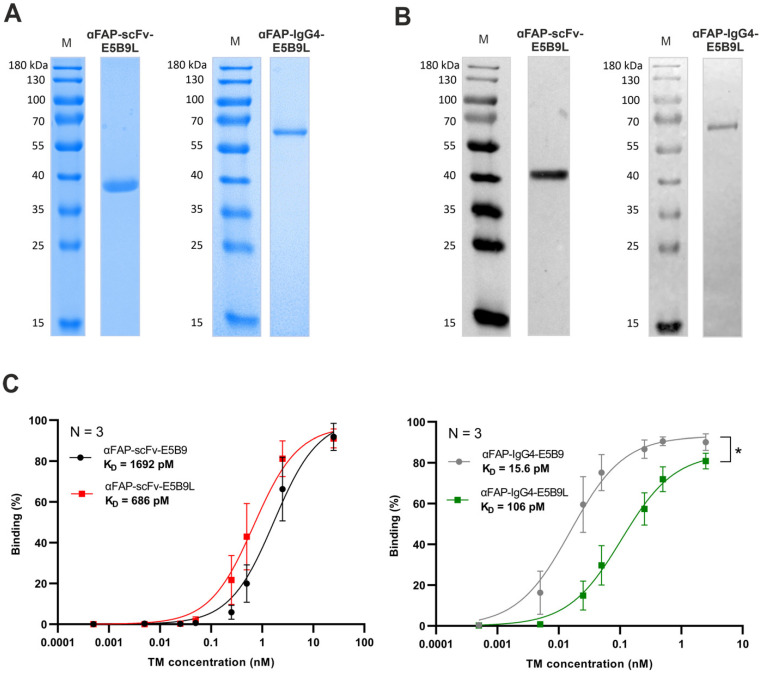
αFAP-E5B9L TMs expression and binding assessment to UniCAR T-cells using flow cytometry. TMs were produced recombinantly by stable producer cell lines and purified from cell culture supernatants using His-tag affinity chromatography. (**A**) Purified TMs were analyzed using SDS-PAGE under reducing conditions followed by Coomassie staining. (**B**) Alternatively, the separated proteins were transferred to nitrocellulose membrane and detected by immunoblotting using anti-penta-His mAb. (**C**) The binding of different concentrations of αFAP TMs to UniCAR T-cells was detected using anti-His antibody and flow cytometry. Statistical analysis was performed using GraphPad Prism 9.0 and statistical significance was determined using two-tailed paired T-test (* *p* ≤ 0.012). M: molecular weight. K_D_: equilibrium dissociation constant.

After successful cloning of αFAP-scFv-E5B9L and αFAP-IgG4-E5B9L TMs in lentiviral vectors, murine 3T3 producer cell lines were stably transduced to express and secrete the TMs. The respective TMs were purified from cell culture supernatant using Ni-NTA affinity chromatography. Isolated TMs were subsequently analyzed by SDS-PAGE under reducing conditions (Figure 3A) followed by Western blotting (Figure 3B). The theoretical native molecular weights calculated for the αFAP-scFv-E5B9L and αFAP-IgG4-E5B9L are approximately 32 kDa and 112 kDa, respectively. After SDS-PAGE and Western blotting, protein bands were visualized with mobilities according to molecular weights of around 40 kDa for the scFv-based TM and 67 kDa for the IgG4-based TM, respectively. As the native IgG4-based TM is a homodimer, the molecule becomes a monomer under reducing conditions. The presence of post-translational modifications (most likely glycosylation) may explain the differences between the theoretical and observed molecular weights of the TMs. Overall, both αFAP-E5B9L TMs were successfully expressed, purified and biochemically characterized, enabling further in vitro assessment for comparison of affinity properties and functionality to the αFAP-E5B9 TMs.

The binding properties of the αFAP-E5B9 and αFAP-E5B9L TMs to UniCAR T-cells were then estimated. UniCAR T-cells were incubated with different concentrations of αFAP-scFv-E5B9, αFAP-scFv-E5B9L, αFAP-IgG4-E5B9 or αFAP-IgG4-E5B9L TMs and the specific binding and binding affinity of each αFAP TM were estimated using flow cytometry. As depicted in Figure 3C, all TMs bind to UniCAR T-cells in a dose-dependent manner with high affinity and apparent equilibrium dissociation constant (K_D_) values varying from the low picomolar to the low nanomolar range. Compared to the αFAP-scFv-E5B9, the αFAP-scFv-E5B9L TM exhibits a 2.5-times higher apparent affinity, with apparent K_D_ values of 686 and 1692 pM, respectively. As expected, all IgG4-based TMs show higher apparent affinity to the UniCAR T-cells than the scFv-based TMs, consistent with the fact that the cross-linking of a bivalent molecule to two targets can result in synergistic interactions that can enhance apparent affinity (i.e., avidity) [66,67]. Unexpectedly, however, the αFAP-IgG4-E5B9 TM has a 6.6-times higher affinity to UniCAR T-cells compared to the αFAP-IgG4-E5B9L TM, with K_D_ values of 15.6 pM and 106 pM, respectively. Interestingly, the binding of the αFAP-E5B9L TMs to FAP^+^ target cells was similar to that of αFAP-E5B9 TMs, demonstrating that the modifications in the epitope regions do not affect the affinity of the TMs to the TAA (Supplementary Figure S1).

### 2.4. Anti-FAP E5B9L TMs Successfully Redirect UniCAR T-Cells to Eradicate FAP^+^ Cells

**Figure 4:**
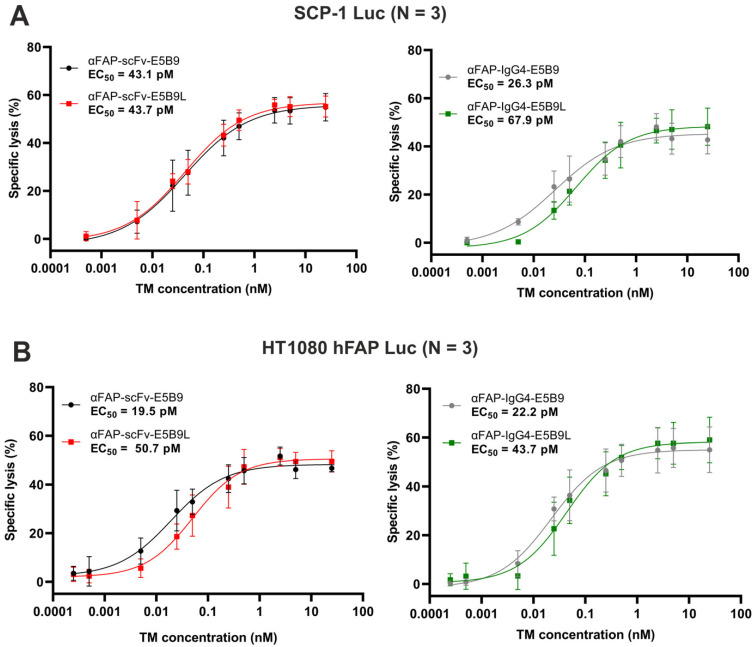
Dose–response curves and effective concentrations of αFAP TMs in the presence of UniCAR T-cells. To determine dose–response curves and EC_50_ of the αFAP TMs, SCP-1 Luc (**A**) or HT1080 hFAP Luc (**B**) cell lines were incubated for 8h with UniCAR T-cells at an E:T ratio of 5:1 in the presence of a range of αFAP TM concentrations. UniCAR T-cell-promoted cytotoxicity was evaluated using luciferase-based assay. Results are shown as mean ± SD of three independent T-cell donors. Killing data were normalized to target cells incubated with UniCAR T-cells in the absence of TM. EC_50_: half maximal effective concentration.

According to the binding studies, the affinities of the four αFAP TMs to the UniCAR T-cells vary in a wide range from around 16 to 1700 pM (700-1700 pM for scFv formats and 16-110 pM for IgG4-based molecules). We thus evaluated the influence of the affinity between the UniCAR epitope and the UniCAR on the potency of UniCAR T-cells. For this purpose, UniCAR T-cells were co-cultured with either SCP-1 Luc (Figure 4A) or HT1080 hFAP Luc (Figure 4B) cells in the absence or presence of increasing concentrations of the αFAP-E5B9 and αFAP-E5B9L TMs. All TMs are able to redirect UniCAR T-cells and kill FAP^+^ cells in a dose-dependent manner in both SCP-1 Luc and HT1080 hFAP Luc models while in the absence of any TM, only minimal killing is observed (Supplementary Figure S2). No significant difference in dose–response curves is observed across TM formats or cell models, with half maximal effective concentrations (EC_50_) values in the low picomolar range between about 19 pM and 67 pM. These data demonstrate that, in spite of the difference in the binding to UniCAR T-cells, αFAP-E5B9 and αFAP-E5B9L TMs allow for a comparable effective and potent redirection of UniCAR T-cells to eliminate FAP^+^ cells. Consequently, the over 100-fold range of affinity from around 16 to 1700 pM has no dramatic effect on the functionality of the UniCAR T-cells.

### 2.5. UniCAR T-Cells Redirected by αFAP-E5B9L TMs Promote the Specific Release of Proinflammatory Cytokines

**Figure 5:**
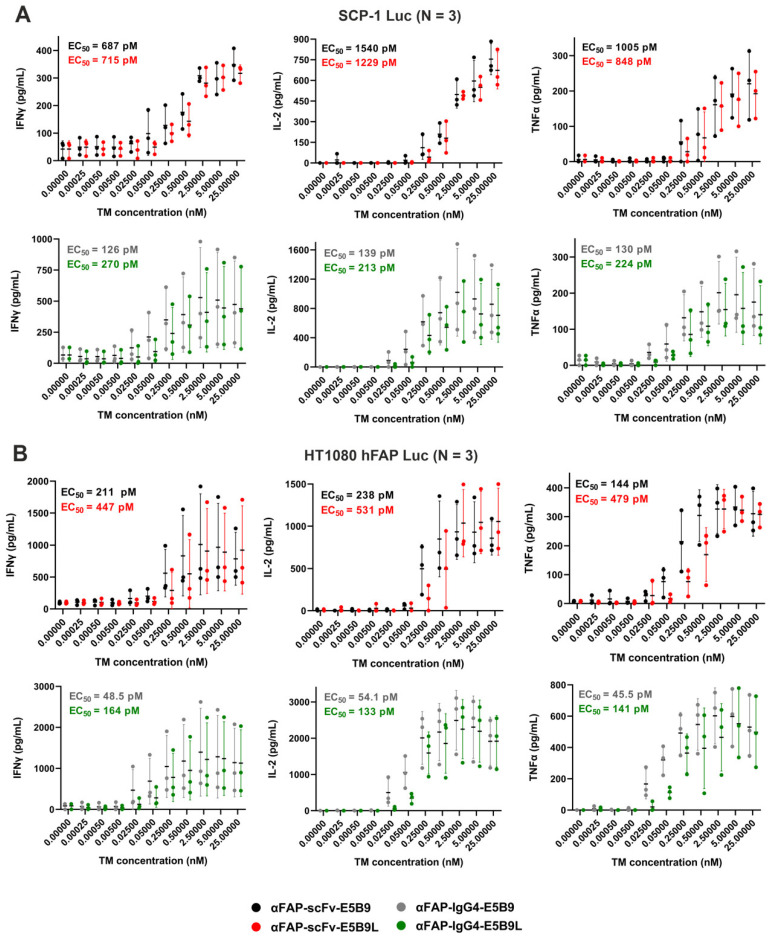
Release of pro-inflammatory cytokines by UniCAR T-cells upon TM cross-linking of effector and target cells. UniCAR T-cells and SCP-1 Luc (**A**) or HT1080 hFAP Luc (**B**) were incubated at an E:T ratio of 5:1 in the presence of different αFAP TM concentrations. After 8h, IFNγ, IL-2 and TNFα concentrations in co-culture supernatants were determined by ELISA. Results are shown as mean ± SD for three independent T-cell donors.

A more sensitive indicator than cell lysis for UniCAR functionality is the estimation of released cytokines. In order to estimate the proinflammatory cytokine release profile of effector cells, UniCAR T-cells were co-cultured with either SCP-1 Luc (Figure 5A) or HT1080 hFAP Luc (Figure 5B) cells in the absence or presence of the αFAP-E5B9 and αFAP-E5B9L TMs. Consistent with the cytotoxic potential of UniCAR T-cells obtained, the secretion of Interferon gamma (IFNγ), Interleukin 2 (IL-2) and Tumor Necrosis Factor alpha (TNFα) increase in a TM dose-dependent manner in both FAP^+^ tumor models and for all TMs. As expected, in the absence of TM, extremely low levels of secreted inflammatory cytokines are detected. Again, no major differences are observed in the cytokine secretion pattern between αFAP-scFv-E5B9 and αFAP-scFv-E5B9L nor between αFAP-IgG4-E5B9 and αFAP-IgG4-E5B9L using the SCP-1 Luc tumor model. In contrast, using the HT1080 hFAP Luc model, there is a 2–3-fold difference in calculated EC_50_ when comparing αFAP-scFv-E5B9 with αFAP-scFv-E5B9L and αFAP-IgG4-E5B9 with αFAP-IgG4-E5B9L. Overall, IgG4-based TMs are associated with lower EC_50_ values for each cytokine and trigger higher maximal amounts of secreted cytokines when compared to scFv-based TMs using both tumor models.

## 3. Discussion

Despite the significant achievements of clinically approved CAR T-cell products, conventional CAR T-cell therapy continues to face various obstacles, including major side effects due to massive CAR T-cell activation, on-target/off-tumor effect and antigen loss of tumor escape variants [15,16,17,18,19,68,69]. The elimination of healthy tissue as side effects becomes of special relevance when the CAR technology is applied for the treatment of autoreactive immune cells as in the case of SLE and primary Sjögren’s syndrome. In order to improve the safety management of CAR T-cell treatment, several groups have focused on modulating the function of conventional CAR T-cells by, e.g., tuning the affinity of the CAR antigen-binding domain [59,62], using different signaling and/or costimulatory domains [70,71]. For better safety and flexibility, others have changed the original rigid structure of CARs and developed universal CAR platforms based on the use of intermediary molecules to redirect CAR T-cells to the target antigen [20,21,22,23,24,25,26,27,28,29,30,31,32,33,34,35,36,37]. In such systems, CAR T-cell activation and functionality directly rely on the presence and amount of the bridging molecule but can also be modulated by selecting different intracellular domains and combinations thereof, refining the CAR antigen-binding domain and modifying the structure and valency of the adaptor molecule to change its affinity to the universal CARs or target antigens. To date, only two groups have shown that modular CAR platforms can be modulated by fine-tuning CAR affinity and/or modifying the structure of associated adaptor molecules [21,24]. In the split, universal and programmable CAR system, Cho et al. have shown that SUPRA CAR T-cell potency depends on the affinity of the CAR for the leucine zipper-based adaptor molecule: the higher the affinity, the greater the potency [21]. In contrast, by employing amphiphile conjugate of the small molecule fluorescein isothiocyanate (FITC) inserted in the membrane of cancer cells to redirect FITC-specific CAR T-cells against tumor cells, Zhang et al. have demonstrated that T-cells engineered with CAR scFvs ranging from low to high affinity (K_D_ between fM and nM) to FITC is associated with similar therapeutic effect in mouse models [24].

In this study, we aimed to understand how the affinity between the extracellular antibody-binding domain of the UniCAR to the peptide epitope of a TM influences the functionality of UniCAR T-cells. For this purpose, we constructed and compared TMs differing with respect to their binding to the UniCAR in a wide range of affinity. In a first step, we estimated the contribution of each aa in the E5B9 epitope. Oligopeptides of 15 aa overlapping by 14 aa representing the whole aa sequence of the nuclear La autoantigen were constructed and analyzed in parallel for binding to the αLa mAb 5B9. Thereby we confirmed the previously determined minimal epitope sequence KPLPEVTDEY [25]. Although not absolutely required for binding, the addition of up to five aa upstream of the minimal epitope strongly improves the binding of the αLa mAb 5B9, resulting in the identification of the elongated epitope named E5B9L. In addition, replacing each of the aa of the E5B9 epitope with one of the 20 standard aa shows that the leucine and the threonine residues are absolutely required for binding. Also, the tyrosine residue cannot be replaced by any aa, with the exception of the aromatic aa phenylalanine, which seems to enhance the binding capability of the αLa mAb 5B9. Based on this detailed knowledge, we decided to construct additional TMs directed to FAP containing the elongated E5B9L epitope instead of the minimal E5B9 epitope. Both scFv- and IgG4-based αFAP-E5B9 TMs were modified and the apparent K_D_ values for the E5B9- and E5B9L-based TMs were estimated by evaluating their binding to UniCAR T-cells. The estimated K_D_ values of the four αFAP TMs ranged from the low picomolar (16 pM) to the nanomolar range (1.7 nM). In spite of this wide range of more than 100-fold, we did not see a dramatic difference with respect to either the lysis of FAP^+^ tumor cells or release of cytokines. The EC_50_ values of all αFAP TMs were in the low picomolar range. Thus, our results are in line with the work conducted by Zhang et al. with the FITC-specific modular CAR platform [24], suggesting that increased affinity may not necessarily be associated with increased potency for certain modular CAR approaches. 

Overall, our data suggest that the binding affinity between the adaptor molecule and the adaptor CAR T-cell is not critical and can vary in a wide range without dramatic effect on the functionality and potency of the adaptor CAR T-cells in vitro. Further in vivo studies to confirm such in vitro data will be the focus of upcoming work that will be published separately.

## 4. Materials and Methods

### 4.1. Cell Lines

Two cell lines, SCP-1 Luc and HT1080 hFAP Luc, were previously generated and used in this study as target cell models [48]. Both cell lines express the firefly luciferase (Luc): SCP-1 Luc endogenously expresses FAP while HT1080 hFAP Luc was genetically engineered for FAP overexpression. Additionally, murine 3T3 cell lines served as the cell model to express previously described and novel anti-FAP (αFAP) TMs used throughout this work. All cell lines were maintained at 37 °C in a humidified atmosphere with 5% CO_2_ and cultured in complete DMEM medium.

### 4.2. Generation and Maintenance of UniCAR T-Cells

Human Peripheral Blood Mononuclear Cells (PBMCs) were obtained from buffy coats of healthy donors (German Red Cross, Dresden) using density centrifugation with Pancoll separating buffer (PanBiotech, Aidenbach, Germany). Pan T-cells were subsequently isolated by negative selection using Pan T-cell isolation kit according to the manufacturer’s instructions (Miltenyi Biotec, Bergisch Gladbach, Germany) and maintained in RPMI complete medium supplemented with IL-2 (50 U/mL) for 3 days. Isolated T-cells were activated using T-Cell TransAct (Miltenyi Biotec) and UniCAR T-cells were generated via lentiviral transduction as previously described [20]. During expansion, T-cells were cultured in TexMACS medium (Miltenyi Biotec) supplemented with human IL-2, IL-7 and IL-15 (Miltenyi Biotec). UniCAR T-cells were cultured in complete RPMI medium without cytokines 24h prior to the experiments. 

### 4.3. Epitope Mapping Studies

The affinity of αLa mAb 5B9 to La-derived peptides was studied as described previously [25]. In addition, PEPperMAP^®^ epitope mappings of αLa mAb 5B9 were performed by the company PEPperPRINT (Heidelberg, Germany). Two La peptide microarrays were generated. On the one hand, the La protein was converted into linear 15 aa peptides with a peptide–peptide overlap of 14 aa. On the other hand, every aa from the wild-type La peptide N_ter_-PSKPLPEVTDEYKND-C_ter_ was replaced by each of the standard 20 aa. For both studies, the peptides generated were printed on a high-density peptide microarray. The La peptide microarrays were then incubated with mouse αLa mAb 5B9 and subsequently stained with anti-mouse IgG (H + L) DyLight680. Fluorescence was acquired with an Innopsys InnoScan 710-IR Microarray Scanner.

### 4.4. Construction, Expression, Purification and Characterization of Recombinant αFAP TMs

The αFAP TMs containing the UniCAR epitope E5B9, αFAP-scFv and αFAP-IgG4, were designed and cloned as described in a previous publication [48]. For clarity, they were herein respectively renamed αFAP-scFv-E5B9 and αFAP-IgG4-E5B9. In this study, the E5B9L peptide variant N_ter_-RRSPSKPLPEVTDEY-C_ter_ was used to construct a novel UniCAR epitope. For the design and cloning of the αFAP TMs containing the E5B9L variant, the previously established αFAP-scFv-E5B9 and αFAP-IgG4-E5B9 vectors were used and the E5B9 epitope sequence was simply replaced by the E5B9L sequence, resulting in the generation of αFAP-scFv-E5B9L and αFAP-IgG4-E5B9L lentiviral vectors (Figure 2B). These vectors were used to produce lentiviral particles encoding the TMs, which were subsequently used to transduce 3T3 cells for stable expression of the αFAP TMs. Secreted TMs were then purified by Ni-NTA affinity chromatography using Ni-NTA agarose (Qiagen, Hilden, Germany) on Poly-Prep Chromatography Columns (Bio-Rad Laboratories, Feldkirchen, Germany). After the supernatant passed through the column, each column was washed once with washing buffer 1 (150 mM NaCl and 10 mM imidazole in PBS) and twice with washing buffer 2 (150 mM NaCl and 20 mM imidazole in PBS). Proteins were eluted twice with elution buffer (150 mM NaCl and 350 mM imidazole in PBS). After dialysis in PBS, the purity and the concentration of the TMs were determined using SDS-PAGE and Western Blot (WB), as detailed in previous publications (e.g., [48]). Briefly, after electrophoresis, gels were either stained with Quick Coomassie Stain (Protein Ark, Rotherham, UK) or transferred to nitrocellulose membrane. His-tagged TMs were detected by a mouse anti–penta-his mAb (Qiagen, Hilden, Germany) and an anti-mouse IgG conjugated with alkaline phosphatase (Dianova, Hamburg, Germany) using 5-bromo-4-chloro-indolyl phosphate (BCIP) (Gerbu Biotechnik GmbH, Gaiberg, Germany).

### 4.5. Binding Assay Using Flow Cytometry

Binding properties and affinities of the αFAP TMs to UniCAR T-cells were determined using flow cytometry. Shortly, 1 × 10^5^ effector cells were incubated for 1 h with increasing concentrations of TMs diluted in staining buffer (PBS/2% FBS). After washing with staining buffer, TM binding was subsequently detected after 30 min incubation using an anti-His antibody (Miltenyi Biotec). All incubation steps were performed at 4 °C in the dark and dead cells were distinguished by adding propidium iodide (PI) (Miltenyi Biotec). Stained cells were analyzed using MACSQuant VYB and MACSQuantify software (Miltenyi Biotec). Binding curves were obtained and K_D_ values were calculated using the nonlinear regression curve fit with GraphPad Prism 9 software (GraphPad Software Inc., Boston, MA, USA).

### 4.6. Luminescence-Based Cytotoxicity Assay

The cytotoxic potential of UniCAR T-cells was analyzed using target cells expressing Luc according to previously published protocols [49]. In short, triplets of 5 × 10^3^ target cells were seeded in 96-well white plates (Greiner Bio-One, Kremsmünster, Austria) and co-cultured with 2.5 × 10^4^ UniCAR T-cells (E:T ratio of 5:1) in the absence or presence of different concentrations αFAP TMs. After 8 h of co-culture, the specific lysis of the target cells was calculated. Data were acquired using the Infinite M200 Pro plate reader (Tecan Trading AG, Männedorf, Switzerland). Dose–response curves were obtained and EC_50_ values were calculated using GraphPad Prism 9 software.

### 4.7. Cytokine-Release Assay

To evaluate the levels of secreted IFNγ, IL-2 and TNFα by UniCAR T-cells, 5 × 10^3^ target cells were seeded in 96-well cell culture plates and cultivated with 2.5 × 10^4^ UniCAR T-cells in the absence or presence of αFAP TMs. Supernatants of these co-culture assays were harvested after 8 h and cytokines were detected using OptEIA Human IFNγ, IL-2 and TNFα ELISA Sets according to the manufacturer´s instructions (BD BioSciences, Heidelberg, Germany). Dose–response curves were obtained and EC_50_ values were calculated using GraphPad Prism 9 software (GraphPad Software Inc.).

## Data Availability

The data presented in this study are available on request from the corresponding author.

## Supplementary Materials

The supporting information can be downloaded at: https://www.mdpi.com/article/10.3390/ijms25137242/s1.

## References

[1] Gross G., Waks T., Eshhar Z. (1989). Expression of immunoglobulin-T-cell receptor chimeric molecules as functional receptors with antibody-type specificity. Proc. Natl. Acad. Sci. USA.

[2] June C.H., O’Connor R.S., Kawalekar O.U., Ghassemi S., Milone M.C. (2018). CAR T cell immunotherapy for human cancer. Science.

[3] Sadelain M. (2016). Chimeric antigen receptors: Driving immunology towards synthetic biology. Curr. Opin. Immunol..

[4] Maher J., Brentjens R.J., Gunset G., Rivière I., Sadelain M. (2002). Human T-lymphocyte cytotoxicity and proliferation directed by a single chimeric TCRζ/CD28 receptor. Nat. Biotechnol..

[5] Harrer D.C., Li S.-S., Kaljanac M., Barden M., Pan H., Abken H. (2023). Fine-tuning the antigen sensitivity of CAR T cells: Emerging strategies and current challenges. Front. Immunol..

[6] Müller F., Taubmann J., Bucci L., Wilhelm A., Bergmann C., Völkl S., Aigner M., Rothe T., Minopoulou I., Tur C. (2024). CD19 CAR T-Cell Therapy in Autoimmune Disease—A Case Series with Follow-up. N. Engl. J. Med..

[7] Mackensen A., Müller F., Mougiakakos D., Böltz S., Wilhelm A., Aigner M., Völkl S., Simon D., Kleyer A., Munoz L. (2022). Anti-CD19 CAR T cell therapy for refractory systemic lupus erythematosus. Nat. Med..

[8] Carney E.F. (2022). Treatment of SLE with anti-CD19 CAR-T cells. Nat. Rev. Nephrol..

[9] Kalos M., Levine B.L., Porter D.L., Katz S., Grupp S.A., Bagg A., June C.H. (2011). T Cells with Chimeric Antigen Receptors Have Potent Antitumor Effects and Can Establish Memory in Patients with Advanced Leukemia. Sci. Transl. Med..

[10] Brentjens R.J., Davila M.L., Riviere I., Park J., Wang X., Cowell L.G., Bartido S., Stefanski J., Taylor C., Olszewska M. (2013). CD19-Targeted T Cells Rapidly Induce Molecular Remissions in Adults with Chemotherapy-Refractory Acute Lymphoblastic Leukemia. Sci. Transl. Med..

[11] Grupp S.A., Kalos M., Barrett D., Aplenc R., Porter D.L., Rheingold S.R., Teachey D.T., Chew A., Hauck B., Wright J.F. (2013). Chimeric Antigen Receptor–Modified T Cells for Acute Lymphoid Leukemia. N. Engl. J. Med..

[12] Lee D.W., Kochenderfer J.N., Stetler-Stevenson M., Cui Y.K., Delbrook C., Feldman S.A., Fry T.J., Orentas R., Sabatino M., Shah N.N. (2015). T cells expressing CD19 chimeric antigen receptors for acute lymphoblastic leukaemia in children and young adults: A phase 1 dose-escalation trial. Lancet.

[13] Johnson P.C., Abramson J.S. (2022). Engineered T Cells: CAR T Cell Therapy and Beyond. Curr. Oncol. Rep..

[14] Zhang X., Zhu L., Zhang H., Chen S., Xiao Y. (2022). CAR-T Cell Therapy in Hematological Malignancies: Current Opportunities and Challenges. Front. Immunol..

[15] Morgan R.A., Yang J.C., Kitano M., Dudley M.E., Laurencot C.M., Rosenberg S.A. (2010). Case Report of a Serious Adverse Event Following the Administration of T Cells Transduced with a Chimeric Antigen Receptor Recognizing ERBB2. Mol. Ther..

[16] Asghar M.S.M., Shah S.M.M.I., Rani A.M., Kazmi S.M., Savul I.S., Ukrani J., Khan F.M., Hasan C.A.M., Rathore N.M., Syed M.M. (2023). Toxicities of CAR T-cell therapy: A review of current literature. Ann. Med. Surg..

[17] Sharma R., Suravarjhula L., Banerjee M., Kumar G., Kumar N. (2023). Chimeric Antigen Receptor T-cell Therapy in Cancer: A Critical Review. Curr. Drug Res. Rev..

[18] Chohan K.L., Siegler E.L., Kenderian S.S. (2023). CAR-T Cell Therapy: The Efficacy and Toxicity Balance. Curr. Hematol. Malig. Rep..

[19] Bove C., Arcangeli S., Falcone L., Camisa B., El Khoury R., Greco B., De Lucia A., Bergamini A., Bondanza A., Ciceri F. (2023). CD4 CAR-T cells targeting CD19 play a key role in exacerbating cytokine release syndrome, while maintaining long-term responses. J. Immunother. Cancer.

[20] Cartellieri M., Feldmann A., Koristka S., Arndt C., Loff S., Ehninger A.V., Von Bonin M., Bejestani E.P., Ehninger G., Bachmann M.P. (2016). Switching CAR T cells on and off: A novel modular platform for retargeting of T cells to AML blasts. Blood Cancer J..

[21] Cho J.H., Collins J.J., Wong W.W. (2018). Universal Chimeric Antigen Receptors for Multiplexed and Logical Control of T Cell Responses. Cell.

[22] Koristka S., Cartellieri M., Feldmann A., Arndt C., Loff S., Michalk I., Aliperta R., von Bonin M., Bornhäuser M., Ehninger A. (2014). Flexible Antigen-Specific Redirection of Human Regulatory T Cells Via a Novel Universal Chimeric Antigen Receptor System. Blood.

[23] Park S., Pascua E., Lindquist K.C., Kimberlin C., Deng X., Mak Y.S.L., Melton Z., Johnson T.O., Lin R., Boldajipour B. (2021). Direct control of CAR T cells through small molecule-regulated antibodies. Nat. Commun..

[24] Zhang A.Q., Hostetler A., Chen L.E., Mukkamala V., Abraham W., Padilla L.T., Wolff A.N., Maiorino L., Backlund C.M., Aung A. (2023). Universal redirection of CAR T cells against solid tumours via membrane-inserted ligands for the CAR. Nat. Biomed. Eng..

[25] Bachmann M. (2019). The UniCAR system: A modular CAR T cell approach to improve the safety of CAR T cells. Immunol. Lett..

[26] Feldmann A., Arndt C., Koristka S., Berndt N., Bergmann R., Bachmann M.P. (2019). Conventional CARs versus modular CARs. Cancer Immunol. Immunother..

[27] Kittel-Boselli E., Soto K.E.G., Loureiro L.R., Hoffmann A., Bergmann R., Arndt C., Koristka S., Mitwasi N., Kegler A., Bartsch T. (2021). Targeting Acute Myeloid Leukemia Using the RevCAR Platform: A Programmable, Switchable and Combinatorial Strategy. Cancers.

[28] Önder C.E., Moustafa-Oglou M., Schröder S.M., Hartkopf A.D., Koch A., Seitz C.M. (2023). Precision Immunotherapy Utilizing Adapter CAR-T Cells (AdCAR-T) in Metastatic Breast Cancer Leads to Target Specific Lysis. Cancers.

[29] Stepanov A.V., Xie J., Zhu Q., Shen Z., Su W., Kuai L., Soll R., Rader C., Shaver G., Douthit L. (2023). Control of the antitumour activity and specificity of CAR T cells via organic adapters covalently tethering the CAR to tumour cells. Nat. Biomed. Eng..

[30] Nixdorf D., Sponheimer M., Berghammer D., Engert F., Bader U., Philipp N., Kazerani M., Straub T., Rohrbacher L., Wange L. (2023). Adapter CAR T cells to counteract T-cell exhaustion and enable flexible targeting in AML. Leukemia.

[31] Borrok M.J., Li Y., Harvilla P.B., Vellalore Maruthachalam B., Tamot N., Prokopowitz C., Chen J., Venkataramani S., Grewal I.S., Ganesan R. (2022). Conduit CAR: Redirecting CAR T-Cell Specificity with A Universal and Adaptable Bispecific Antibody Platform. Cancer Res. Commun..

[32] Werchau N., Kotter B., Criado-Moronati E., Gosselink A., Cordes N., Lock D., Lennartz S., Kolbe C., Winter N., Teppert K. (2022). Combined targeting of soluble latent TGF-ß and a solid tumor-associated antigen with adapter CAR T cells. OncoImmunology.

[33] Atar D., Mast A.-S., Scheuermann S., Ruoff L., Seitz C.M., Schlegel P. (2022). Adapter CAR T Cell Therapy for the Treatment of B-Lineage Lymphomas. Biomedicines.

[34] Seitz C.M., Mittelstaet J., Atar D., Hau J., Reiter S., Illi C., Kieble V., Engert F., Drees B., Bender G. (2021). Novel adapter CAR-T cell technology for precisely controllable multiplex cancer targeting. OncoImmunology.

[35] McCue A.C., Yao Z., Kuhlman B. (2022). Advances in modular control of CAR-T therapy with adapter-mediated CARs. Adv. Drug Deliv. Rev..

[36] Landgraf K.E., Williams S.R., Steiger D., Gebhart D., Lok S., Martin D.W., Roybal K.T., Kim K.C. (2020). convertibleCARs: A chimeric antigen receptor system for flexible control of activity and antigen targeting. Commun. Biol..

[37] Minutolo N.G., Hollander E.E., Powell D.J.J. (2019). The Emergence of Universal Immune Receptor T Cell Therapy for Cancer. Front. Oncol..

[38] Peng H., Nerreter T., Mestermann K., Wachter J., Chang J., Hudecek M., Rader C. (2022). ROR1-targeting switchable CAR-T cells for cancer therapy. Oncogene.

[39] Bachmann M., Tröster H., Bartsch H., Grölz D. (1996). A Frame Shift Mutation in a Hot Spot Region of the Nuclear Autoantigen La (SS-B). J. Autoimmun..

[40] Kremerskothen J., Nettermann M., op de Bekke A., Bachmann M., Brosius J. (1998). Identification of human autoantigen La/SS-B as BC1/BC200 RNA-binding protein. DNA Cell Biol..

[41] Tran H.B., Ohlsson M., Beroukas D., Hiscock J., Bradley J., Buyon J.P., Gordon T.P. (2002). Subcellular redistribution of La/SSB autoantigen during physiologic apoptosis in the fetal mouse heart and conduction system. Arthritis Rheum..

[42] Carmo-Fonseca M., Pfeifer K., Schröder H.C., Vaz M., Fonseca J., Müller W.E., Bachmann M. (1989). Identification of La ribonucleoproteins as a component of interchromatin granules. Exp. Cell Res..

[43] Topfer F., Gordon T., McCluskey J. (1993). Characterization of the mouse autoantigen La (SS-B). Identification of conserved RNA-binding motifs, a putative ATP binding site and reactivity of recombinant protein with poly(U) and human autoantibodies. J. Immunol..

[44] Chan E.K.L., Sullivan K.F., Fox R.I., Tan E.M. (1989). Sjögren’s syndrome nuclear antigen B (La): cDNA cloning, structural domains, and autoepitopes. J. Autoimmun..

[45] Yiannaki E.E., Tzioufas A.G., Bachmann M., Hantoumi J., Tsikaris V., Sakarellos-Daitsiotis M., Sakarellos C., Moutsopoulos H.M. (1998). The value of synthetic linear epitope analogues of La/SSB for the detection of autoantibodies to La/SSB; specificity, sensitivity and comparison of methods. Clin. Exp. Immunol..

[46] Arndt C., Feldmann A., Koristka S., Schäfer M., Bergmann R., Mitwasi N., Berndt N., Bachmann D., Kegler A., Schmitz M. (2019). A theranostic PSMA ligand for PET imaging and retargeting of T cells expressing the universal chimeric antigen receptor UniCAR. OncoImmunology.

[47] Albert S., Arndt C., Feldmann A., Bergmann R., Bachmann D., Koristka S., Ludwig F., Ziller-Walter P., Kegler A., Gärtner S. (2017). A novel nanobody-based target module for retargeting of T lymphocytes to EGFR-expressing cancer cells via the modular UniCAR platform. OncoImmunology.

[48] Loureiro L.R., Hoffmann L., Neuber C., Rupp L., Arndt C., Kegler A., Kubeil M., Hagemeyer C.E., Stephan H., Schmitz M. (2023). Immunotheranostic target modules for imaging and navigation of UniCAR T-cells to strike FAP-expressing cells and the tumor microenvironment. J. Exp. Clin. Cancer Res..

[49] Loureiro L.R., Feldmann A., Bergmann R., Koristka S., Berndt N., Máthé D., Hegedüs N., Szigeti K., Videira P.A., Bachmann M. (2020). Extended half-life target module for sustainable UniCAR T-cell treatment of STn-expressing cancers. J. Exp. Clin. Cancer Res..

[50] Bouquet L., Bôle-Richard E., Warda W., Neto Da Rocha M., Trad R., Nicod C., Haderbache R., Genin D., Ferrand C., Deschamps M. (2023). RapaCaspase-9-based suicide gene applied to the safety of IL-1RAP CAR-T cells. Gene Ther..

[51] Amatya C., Pegues M.A., Lam N., Vanasse D., Geldres C., Choi S., Hewitt S.M., Feldman S.A., Kochenderfer J.N. (2020). Development of CAR T Cells Expressing a Suicide Gene Plus a Chimeric Antigen Receptor Targeting Signaling Lymphocytic-Activation Molecule F7. Mol. Ther..

[52] Casucci M., Falcone L., Camisa B., Norelli M., Porcellini S., Stornaiuolo A., Ciceri F., Traversari C., Bordignon C., Bonini C. (2018). Extracellular NGFR Spacers Allow Efficient Tracking and Enrichment of Fully Functional CAR-T Cells Co-Expressing a Suicide Gene. Front. Immunol..

[53] Thomas S., Abken H. (2023). CAR T cell therapy becomes CHIC: “cytokine help intensified CAR” T cells. Front. Immunol..

[54] Hombach A., Barden M., Hannappel L., Chmielewski M., Rappl G., Sachinidis A., Abken H. (2021). IL12 integrated into the CAR exodomain converts CD8+ T cells to poly-functional NK-like cells with superior killing of antigen-loss tumors. Mol. Ther..

[55] Gross G., Carmi Y., Abken H. (2022). Editorial: Implementing Logic Gates in Adoptive Cell Therapy. Front. Immunol..

[56] Mestermann K., Giavridis T., Weber J., Rydzek J., Frenz S., Nerreter T., Mades A., Sadelain M., Einsele H., Hudecek M. (2019). The tyrosine kinase inhibitor dasatinib acts as a pharmacologic on/off switch for CAR T cells. Sci. Transl. Med..

[57] Hudecek M., Lupo-Stanghellini M.T., Kosasih P.L., Sommermeyer D., Jensen M.C., Rader C., Riddell S.R. (2013). Receptor Affinity and Extracellular Domain Modifications Affect Tumor Recognition by ROR1-Specific Chimeric Antigen Receptor T Cells. Clin. Cancer Res..

[58] Carnevale J., Shifrut E., Kale N., Nyberg W.A., Blaeschke F., Chen Y.Y., Li Z., Bapat S.P., Diolaiti M.E., O’leary P. (2022). RASA2 ablation in T cells boosts antigen sensitivity and long-term function. Nature.

[59] Celichowski P., Turi M., Charvátová S., Radhakrishnan D., Feizi N., Chyra Z., Šimíček M., Jelínek T., Bago J.R., Hájek R. (2023). Tuning CARs: Recent advances in modulating chimeric antigen receptor (CAR) T cell activity for improved safety, efficacy, and flexibility. J. Transl. Med..

[60] Duan H., Jiang Z., Chen L., Bai X., Cai H., Yang X., Huang H. (2023). TSHR-based chimeric antigen receptor T cell specifically deplete auto-reactive B lymphocytes for treatment of autoimmune thyroid disease. Int. Immunopharmacol..

[61] Liu X., Jiang S., Fang C., Yang S., Olalere D., Pequignot E.C., Cogdill A.P., Li N., Ramones M., Granda B. (2015). Affinity-Tuned ErbB2 or EGFR Chimeric Antigen Receptor T Cells Exhibit an Increased Therapeutic Index against Tumors in Mice. Cancer Res..

[62] Mao R., Kong W., He Y. (2022). The affinity of antigen-binding domain on the antitumor efficacy of CAR T cells: Moderate is better. Front. Immunol..

[63] Meyer J.-E., Loff S., Dietrich J., Spehr J., Jiménez G.J., von Bonin M., Ehninger G., Cartellieri M., Ehninger A. (2021). Evaluation of switch-mediated costimulation in trans on universal CAR-T cells (UniCAR) targeting CD123-positive AML. OncoImmunology.

[64] Koristka S., Cartellieri M., Arndt C., Bippes C.C., Feldmann A., Michalk I., Wiefel K., Stamova S., Schmitz M., Ehninger G. (2013). Retargeting of regulatory T cells to surface-inducible autoantigen La/SS-B. J. Autoimmun..

[65] Wermke M., Kraus S., Ehninger A., Bargou R.C., Goebeler M.E., Middeke J.M., Kreissig C., von Bonin M., Koedam J., Pehl M. (2021). Proof of concept for a rapidly switchable universal CAR-T platform with UniCAR-T-CD123 in relapsed/refractory AML. Blood.

[66] Vauquelin G., Charlton S.J. (2013). Exploring avidity: Understanding the potential gains in functional affinity and target residence time of bivalent and heterobivalent ligands. Br. J. Pharmacol..

[67] Samir P., Kanneganti T.-D. (2019). Hidden Aspects of Valency in Immune System Regulation. Trends Immunol..

[68] Schubert M.-L., Schmitt M., Wang L., Ramos C., Jordan K., Müller-Tidow C., Dreger P. (2020). Side-effect management of chimeric antigen receptor (CAR) T-cell therapy. Ann. Oncol..

[69] Sterner R.C., Sterner R.M. (2021). CAR-T cell therapy: Current limitations and potential strategies. Blood Cancer J..

[70] Honikel M.M., Olejniczak S.H. (2022). Co-Stimulatory Receptor Signaling in CAR-T Cells. Biomolecules.

[71] Meng X., Jing R., Qian L., Zhou C., Sun J. (2020). Engineering Cytoplasmic Signaling of CD28ζ CARs for Improved Therapeutic Functions. Front. Immunol..

