# Supplementary material for: UniCAR T-Cell Potency—A Matter of Affinity between Adaptor Molecules and Adaptor CAR T-Cells?
Source: Int J Mol Sci. 2024 Jun 30;25(13):7242. doi: 10.3390/ijms25137242 (PMC11241561; doi:10.3390/ijms25137242)
Supplement: Supplementary file 1 [file ijms-25-07242-s001.zip › ijms-3061363-supplementary.pdf]

UniCAR T-Cell Potency—A Matter of Affinity between Adaptor Molecules and Adaptor CAR T-Cells?

Hugo Boutier [1,†], Liliana R. Loureiro [1,†], Lydia Hoffmann [1], Claudia Arndt [1,2], Tabea Bartsch [1], Anja Feldmann [1,3,4,5,*] and Michael P. Bachmann [1,3,4,5,*]

Supplementary material

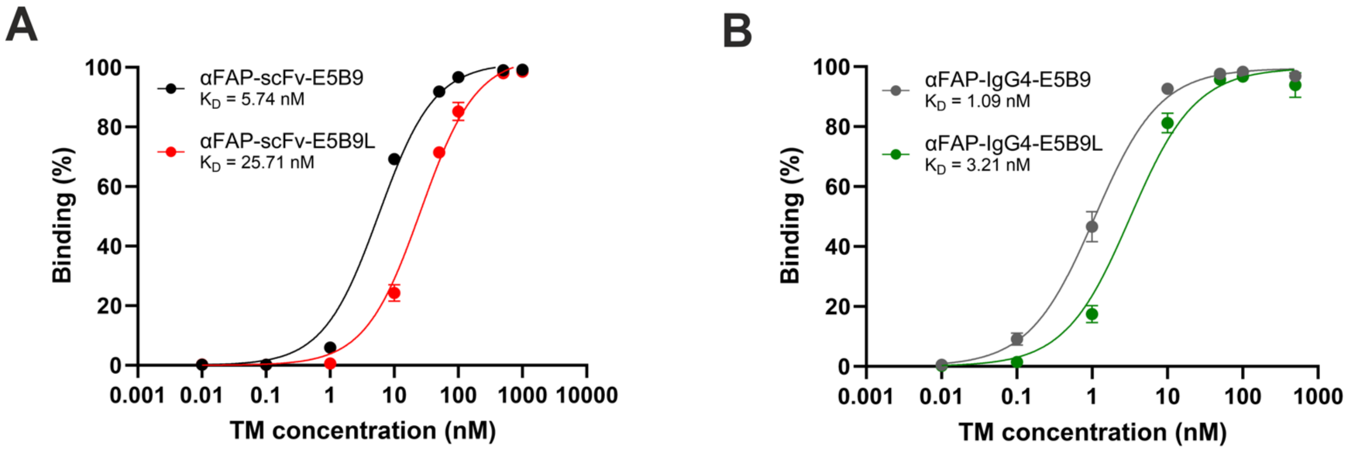

Supplementary Figure S1. Binding assessment of αFAP-E5B9L TMs to FAP+ target cells using flow cytometry. The binding of different concentrations of scFv- (**A**) and IgG4-based (**B**) αFAP TMs to HT1080 hFAP Luc was detected using αLa mAb 5B9 and flow cytometry.

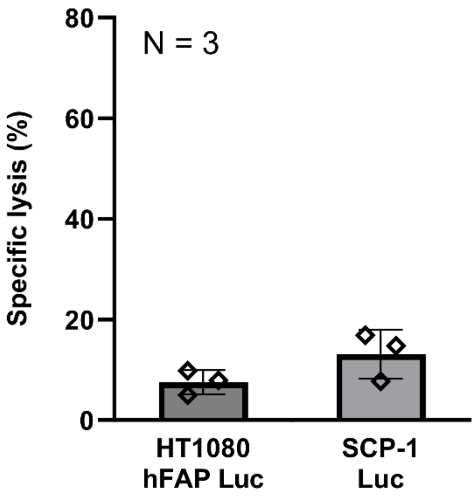

Supplementary Figure S2. Evaluation of the cytotoxic potential of UniCAR T-cells in absence of αFAP TMs. HT1080 hFAP Luc or SCP-1 Luc cell lines were incubated with UniCAR T-cells at an E:T ratio of 5:1 in the absence of αFAP TMs for 8h. The killing was evaluated using luciferase-based assay. Results are shown as mean ± SD of three independent T-cell donors.
